# Palliative Irradiation With CyberKnife and Quad Shot Regimen for an Elderly Patient With Lower Lip Cancer

**DOI:** 10.7759/cureus.83364

**Published:** 2025-05-02

**Authors:** Naomi I Kanno, Yohei Sakurai, Hiromi Hirohata, Shohei Takaoka, Kenji Yamagata

**Affiliations:** 1 Oral and Maxillofacial Surgery, Institute of Medicine, University of Tsukuba, Ibaraki, JPN; 2 Radiation Oncology, Institute of Medicine, University of Tsukuba, Ibaraki, JPN; 3 Oral and Maxillofacial Surgery, Tsukuba Central Hospital, Ibaraki, JPN

**Keywords:** cyberknife, head and neck cancer, oral cancer, palliative radiotherapy, quad shot

## Abstract

A 91-year-old woman noticed a mass on her lower lip and was diagnosed with stage II squamous cell carcinoma (cT2N0M0 as per TNM staging). Due to her advanced age, she and her family declined surgery and opted for radiotherapy (RT) alone (76.0 Gy/38 fractions). The tumor showed a partial response, leading to salvage surgery. Over the next three years, the primary tumor recurred twice, but both recurrences were successfully resected, with no further recurrence afterward. However, 15 months after the last recurrence, she developed submandibular lymph node metastasis and underwent stereotactic radiotherapy (30 Gy/5 fractions) using the CyberKnife, achieving a complete response. Subsequently, multiple metastases appeared in both cervical lymph nodes. She then received intensity-modulated radiotherapy using the Quad-shot method, a type of hypofractionated radiotherapy (44.4 Gy/12 fractions in three cycles). Although a temporary complete response was achieved, cervical lymph node recurrence was subsequently observed. She passed away at the age of 97, six months after the final RT. Significantly, she maintained a good quality of life without significant pain or skin metastases and passed away peacefully at home.

## Introduction

End-stage locally advanced or recurrent/metastatic head and neck cancer can lead to severe complications, including airway obstruction, pain, massive bleeding, and tissue destruction, depending on the tumor’s location. These conditions can rapidly worsen, leading to poor outcomes or significantly reducing quality of life (QOL). Recently, advances in drug therapy, including the advent of molecular-targeted drugs and immune checkpoint inhibitors, have significantly expanded treatment options for locally advanced or recurrent/metastatic head and neck cancer, contributing to extended overall survival and improved outcomes [1]. However, as elderly or frail patients cannot tolerate aggressive treatment with surgery or drug therapy, palliative radiotherapy (RT) that takes into consideration the duration and intensity of treatment is extremely beneficial [2]. Even so, no standardized dose or irradiation method has been established for palliative RT in oral cancer, and comprehensive judgment based on the condition of the patient is necessary.

Recent technological advances have made it possible to use stereotactic radiation therapy (SRT) for recurrent tumors. This has resulted in high local control rates and low toxicity, as well as reduced radiation doses to critical organs. The efficacy and safety of SRT using CyberKnife have been reported as a salvage re-irradiation therapy for recurrent head and neck tumors. In particular, Kobayashi et al. [3] demonstrated the efficacy and safety of SRT for salvage re-irradiation of lymph node recurrence within the radiation field. The Quad-shot (QS) regimen is a phase II trial of RT conducted by the Radiation Therapy Oncology Group in the 1980s for pelvic malignancies. It was administered twice daily for two days at a fractionated dose of 3.7 Gy (14.8 Gy per cycle), repeated for three cycles at intervals of three to six weeks. This regimen was later applied to palliative treatment for head and neck cancer (HNC) and is currently recommended in the National Comprehensive Cancer Network (NCCN) guidelines as a palliative radiation therapy for HNC, using three-dimensional conformal radiation therapy (3D-CRT) or intensity-modulated radiotherapy (IMRT) [4].

In this report, we describe the case of an elderly patient with lower lip cancer who underwent palliative RT using SRT with a CyberKnife and IMRT using the QS method, which is a type of hypofractionated radiotherapy.

## Case presentation

**Figure 1:**
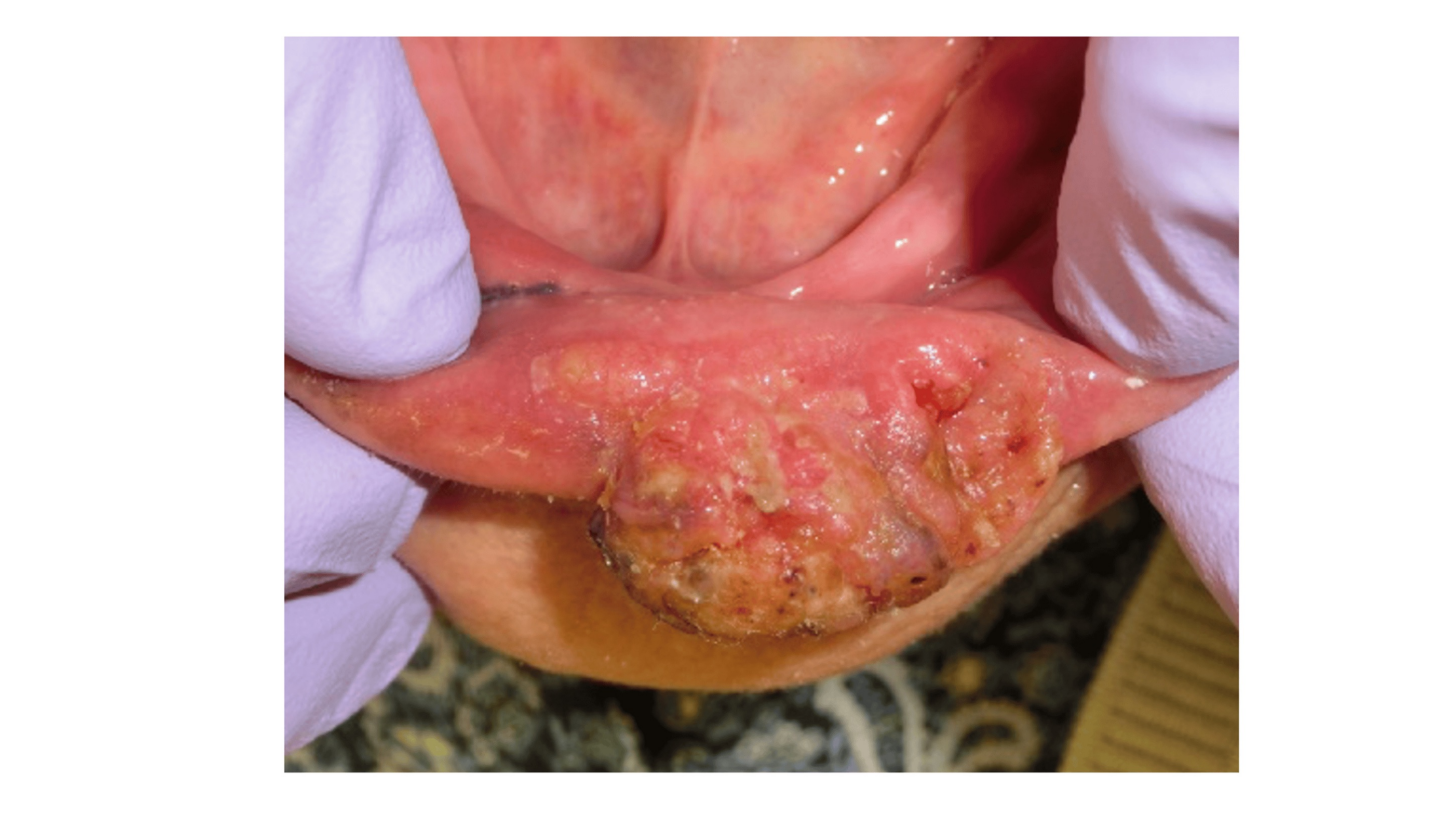
Oral findings at initial examination. A firm mass with a central ulcer measuring 16 × 26 mm was observed on the lower left lip.

A 91-year-old woman was referred to the Department of Oral and Maxillofacial Surgery at the University of Tsukuba Hospital with a progressively enlarging mass on her lower lip for approximately six months. Her medical history included myocardial infarction and osteoporosis. On intraoral examination, a firm, ulcerated mass measuring 16 × 26 mm was observed on the left lower lip (Figure 1).

**Figure 2:**
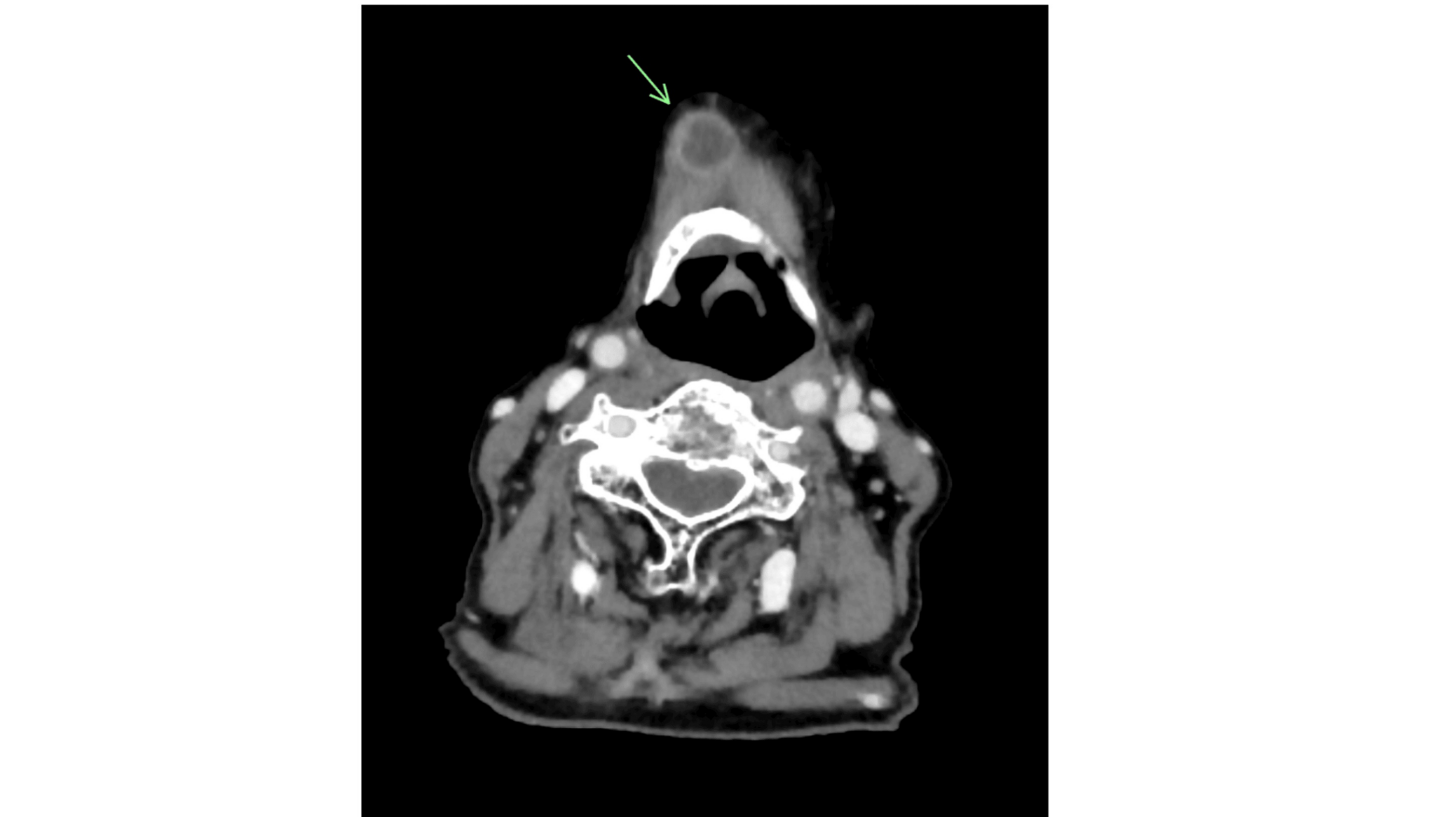
Computed tomography scan showing metastasis in a submental lymph node. Although the primary lesion did not recur, swelling of a submental lymph node with internal necrosis indicated metastasis.

**Figure 3:**
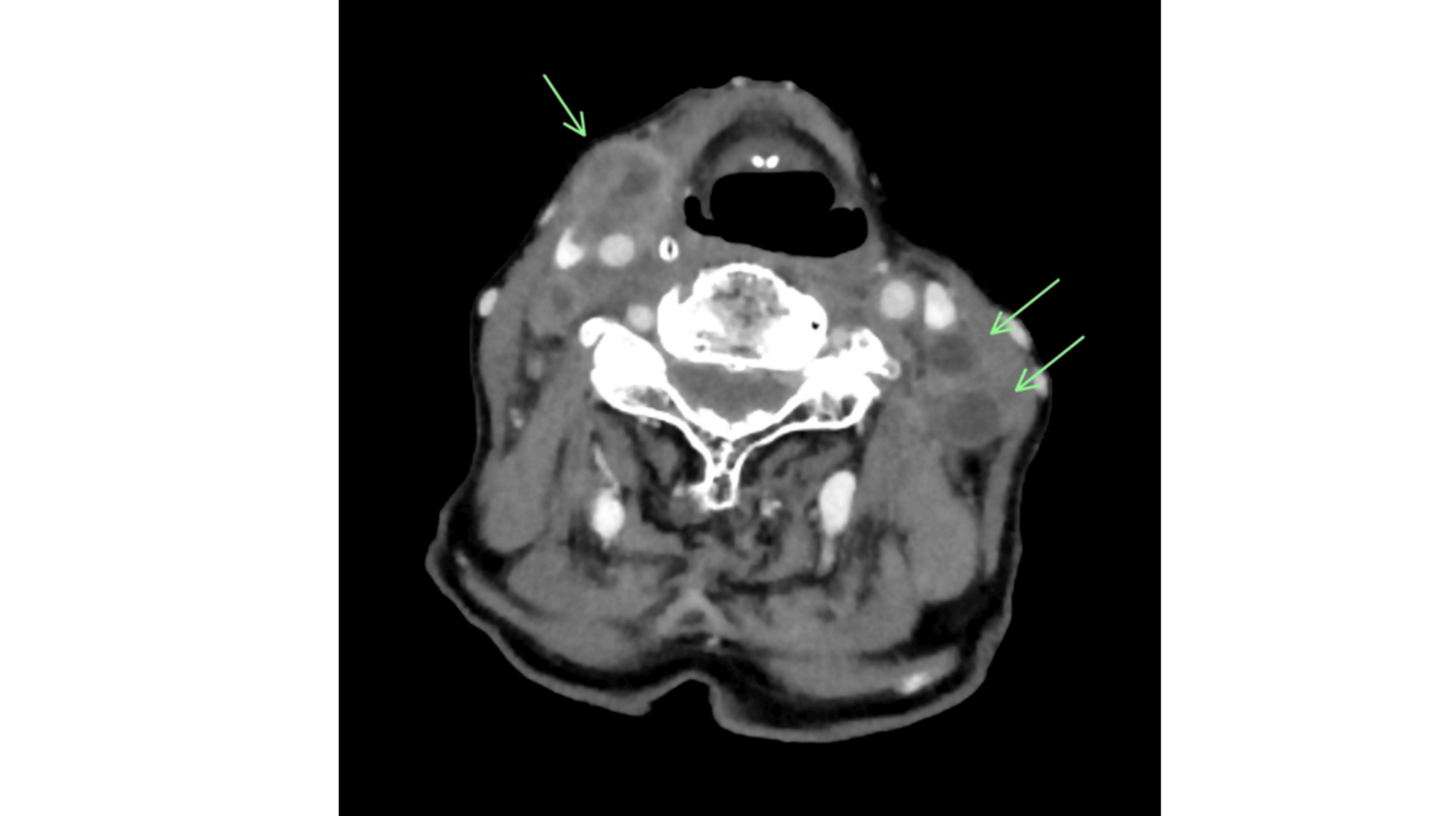
Computed tomography findings post-stereotactic radiotherapy. A complete response was achieved, with resolution of the submental lymph node metastasis; however, bilateral cervical lymph node enlargement and metastases were observed.

Extraoral examination showed a symmetrical facial appearance without submandibular or cervical swelling. Contrast-enhanced computed tomography (CT) revealed an 18 × 14 × 12 mm exophytic, broad-based mass near the midline of the lower lip, with no significant cervical lymph node involvement. Contrast-enhanced magnetic resonance (MR) imaging showed the lesion as mildly hyperintense on T2-weighted images and hypointense on T1-weighted images. Histopathological examination of a biopsy specimen confirmed squamous cell carcinoma, leading to a clinical diagnosis of left lower lip cancer (cT2N0M0; Stage Ⅱ). The patient had a WHO performance status of 0 and was deemed operable. However, she and her family declined surgery due to her advancing age, opting instead for radical RT (3D-CRT, electron beam; 76.0 Gy/38 fractions). Imaging assessment using the Response Evaluation Criteria in Solid Tumors indicated a partial response to RT, and salvage surgery for resection of the remaining tumor was performed. The histopathological diagnosis was squamous cell carcinoma, and resection margins were negative. Three years after the initial treatment, the patient experienced her first recurrence, followed by a second recurrence six months later. Both were successfully resected, and no local recurrence occurred for the remainder of her life. Fifteen months after her last surgery, she developed a submental lymph node metastasis (Figure 2) and underwent CyberKnife-based SRT (30 Gy/5 fractions) as a possible retreatment option, achieving a complete response. Subsequently, multiple bilateral cervical lymph node metastases developed (Figure 3).

Eleven months later, given her performance status of 1, she was treated with palliative RT using a hypofractionated irradiation method (QS: 14.8 Gy/4 fractions per cycle, repeated for three cycles; total dose: 44.4 Gy/12 fractions). The treatment was administered as an inpatient, with each cycle consisting of four fractions over two days (3.7 Gy/fractions; 14.8 Gy/4 fractions), with a total of three cycles (44.4 Gy in total) every four to five weeks. The only adverse event observed was grade 1 dermatitis. A temporary complete response was achieved, but cervical lymph node metastasis recurred again. The patient passed away at the age of 97, six months after the final irradiation, due to disease progression. In selecting a treatment method, as RT was used as the initial treatment, the available dose and dose distribution were examined, and considering the possibility for the patient to be unable to undergo long-term treatment due to her advanced age, it was decided at a conference involving the radiation oncologist, the otolaryngologist/head and neck surgeon, and the oral surgeon. There were no other acute or delayed toxicity during all RT periods (initial RT, CyberKnife-based SRT, and QS), and no complaints of pain until death, except in the perioperative period. Significantly, she maintained a good QOL without pain or cervical skin metastasis, was able to eat with her family until a few days before her death, and passed away peacefully at home.

## Discussion

Patients with HNC, except those with only distant metastases, often experience the terminal stage of their disease along with local tumor progression. As they observe visible tumor growth, they become increasingly aware of their deteriorating condition, leading to immense psychological distress. In advanced head and neck cancer, skin invasion and tissue destruction can cause bleeding, pain, infection, and fluid oozing, resulting in severe functional impairment and a significant decline in QOL. Additionally, the need for continuous local treatment makes end-of-life care more challenging for these patients compared to those with cancer in other organs [2]. Recently, palliative irradiation has been recognized as the standard treatment for brain and bone metastases [5,6]. However, no universal guidelines exist for palliative RT in advanced HNC. Therefore, treatment decisions must be individualized, considering various patient-specific factors. Unlike curative RT, which aims to eradicate cancer, the primary goal of palliative RT is to alleviate symptoms while minimizing adverse events.

Several studies have demonstrated the effectiveness of CyberKnife-based palliative RT for metastatic cervical lymph nodes in HNC. Yamazaki et al. [7] reported on the use of CyberKnife in patients with carotid blowout syndrome in pharyngeal cancer, identifying risk factors, and suggesting the potential for selective treatment options. Kobayashi et al. [3] evaluated 16 cases with 55 recurrent lymph node metastases treated with CyberKnife, reporting a one-year local control rate of 81% and an overall survival rate of 71%. CyberKnife offers the advantage of treatment with a small irradiation field [7] due to its high setup accuracy [8], enabling the delivery of high doses to small targets while minimizing exposure to surrounding organs at risk [3].

Another approach, the QS method, is a form of hypofractionated RT used for palliative treatment. It has few side effects and requires fewer treatment days, being effective for both palliative and local control. Toya et al. [9] analyzed 105 patients, including 13 (12%) patients receiving adjuvant systemic therapy, with HNC who received at least one cycle of the QS regimen (14.8 Gy in four fractions at least six hours apart, repeated up to three times every three to six weeks). Among them, 98 (93%) patients showed an overall response (tumor response or symptom relief), with a response observed in all patients who completed three cycles. Dan et al. [10] investigated 166 patients who underwent the QS regimen as a last-line palliative therapy following prior RT. They reported an overall palliative response rate of 66%, symptom improvement in 60% of patients, and a grade 3 toxicity rate of 10.8% (n = 18), with no cases of grade 4 or 5 toxicity. Upadhyay et al. [11] compared the outcomes of QS with or without concurrent immune checkpoint inhibitors in the palliative treatment of HNC. The combination of QS with concurrent ICIs was well tolerated and significantly improved local control compared to QS alone, with a median overall survival of 9.4 months, which was favorably comparable to historical controls for QS-treated patients. Additionally, Iqbal et al. [12] studied IMRT hypofractionated RT (25 Gy in five fractions) for palliative treatment of HNC. They reported a high treatment completion rate (94%) with good efficacy and tolerability, as 95% of patients experienced no serious treatment-related toxicity. Notably, 63% of the patients in their study had a performance status of 2 or higher. While surgery and chemotherapy are generally limited to patients with a performance status of 0 or 1, RT remains a viable option for those with a poorer performance status, depending on the treatment regimen [3,13].

In our case, the patient was deemed unfit for surgery due to advanced age, and systemic therapy was also contraindicated for the same reason. Generally, concurrent systemic therapy and RT or RT alone are considered for unresectable cases [14]. However, with the advent of molecular-targeted drugs [15] and immune checkpoint inhibitors [16], treatment options have expanded for patients with an adequate performance status. In cases where performance status declines due to recurrence or metastasis, systemic therapy and RT may no longer be feasible, leaving RT alone as the only effective treatment option.

The limitations of this report include the small sample size (only one case to date) and the inability to conduct a prospective follow-up survey.

## Conclusions

We reported a case of palliative RT using SRT with a CyberKnife and IMRT with the QS method, a type of hypofractionated RT, in an elderly patient with advanced lower lip cancer. Despite her disease, local and cervical metastatic lesions were effectively controlled, allowing her to live peacefully with her family until the end, without pain or a significant decline in QOL. While no standardized guidelines exist for palliative RT in advanced HNC, SRT and the QS regimen represent viable treatment options with low toxicity and fewer treatment days, which should be considered based on individual patient needs.
